# Adult Mental Health Associated with Adverse and Positive Childhood Experiences Among 1^st^ and 2^nd^ Generation Asian Americans

**DOI:** 10.1007/s11606-024-09186-8

**Published:** 2024-10-31

**Authors:** Jihoon Jang, Gilbert Gonzales

**Affiliations:** 1 Vanderbilt University School of Medicine, 1161 21stAve S #D3300, Nashville, TN USA; 2 Department of Medicine, Health, and Society, Vanderbilt University, Nashville, TN USA

**Keywords:** adverse childhood experiences, positive childhood experiences, adult mental health, health disparities, Asian Americans

## Abstract

**Background:**

Adverse childhood experiences (ACEs) and positive childhood experiences (PCEs) impact adult health. However, differences in ACEs, PCEs, and mental health have not been extensively studied among Asian Americans.

**Objective:**

To examine the association between childhood experiences and adult mental health in first and second generation Asian Americans.

**Design:**

This study used data from the 2021-2022 California Health Interview Survey (CHIS), an address-based sampling of noninstitutionalized Californians conducted online or by phone.

**Participants:**

Asian American respondents aged 18-65 years.

**Exposure:**

Fifteen different ACEs and seven different PCEs.

**Main Measures:**

Adjusted prevalence ratios (aPR) of severe psychological distress for each generation. Survey weights were applied to all analyses for population-based representation.

**Key Results:**

5,744 Asian Americans (48.0% male, 16.4% aged 18-25) were included in the current study. We found that second generation Asian Americans experienced a greater prevalence of ACEs (65.4% reported ≥1 ACE vs 47.5% in first generation Asian Americans) and lower prevalence of PCEs (32.1% reported ≤2 PCEs vs 22.6% in first generation Asian Americans). Second generation Asian Americans were more likely to report ≥4 ACEs (aPR, 1.46; 95% CI, 1.13 to 1.88) and ≤2 PCEs (aPR, 1.51; 95% CI, 1.29 to 1.78) relative to first generation Asian Americans. Second generation Asian Americans with ≥4 ACEs or ≤2 PCEs were more likely to report severe psychological distress (aPR, 2.54; 95% CI, 1.55 to 4.17 and aPR, 1.48; 95% CI, 1.03 to 2.13, respectively) relative to first generation Asian Americans. When examining ACEs and PCEs individually, domestic, physical, and verbal abuse; divorce; racism; and lacking support systems were significantly associated with severe psychological distress in second generation Asian Americans.

**Conclusions:**

Second generation Asian Americans are more likely to experience more ACEs, fewer PCEs, and poorer mental health as a result. Our study indicates that physicians should screen for childhood experiences and leverage trauma-informed care among Asian American subpopulations.

## INTRODUCTION

Adverse childhood experiences (ACEs) were first defined by Felitti et al. in 1998 and grouped under two major domains: household dysfunction and abuse.^1^ The definition was expanded by Finkelhor et al. in 2013 to include experiences of peer rejection, neighborhood violence, and socioeconomic hardship.^2^ Studies have shown that ACEs are associated with poor adult mental health.^1,3–5^ ACEs and their effects are widely prevalent; more than 60% of Americans report experiencing at least 1 ACE,^6^ and the Centers for Disease Control and Prevention (CDC) estimate that ACEs contribute to >21 million cases of depression annually.^7^ The association between ACEs and mental health is thought to be mediated by chronic stress in childhood that dysregulates neurodevelopment^8–10^ and the neuroendocrine axis,^11^ activates the immune system,^12,13^ and alters epigenetics.^14–16^

In contrast, positive childhood experiences (PCEs) are healthy childhood relationships that individuals share with family and peers,^17^ and are measured using validated tools like the benevolent childhood experiences (BCEs) scale^18^ or the seven-point PCE scale adapted from Child and Youth Resilience Measure-28.^17,19^ Some studies demonstrate that PCEs improve adult mental health by attenuating the effect of ACEs,^18,20,21^ while others suggest that PCEs improve mental health regardless of ACEs.^17,21–23^ However, limited studies have investigated the opposite: does lacking PCEs lead to poorer adult mental health?

While racial disparities in ACEs and PCEs are well-documented,^24,25^ few studies have focused solely on Asian Americans^26^ despite being the fastest growing demographic in the US.^27^ Asian Americans are often homogenized in the literature, but studies have shown that differences in the prevalence of mental health disorders between first and second generation Asian American immigrants exist.^28–31^ However, no studies have specifically looked at intergenerational differences in the association between ACEs, PCEs, and adult mental health within Asian Americans. Our study aims to fill in this knowledge gap by analyzing a largescale, representative survey to estimate associations between ACEs, PCEs, and adult psychological distress among first generation and second generation Asian Americans.

## METHODS

### Data Source

This study utilized publicly available data from the 2021 and 2022 California Health Interview Survey (CHIS), administered annually by the UCLA Center for Health Policy Research to collect data on the health of Californians (the largest US state by population).^32,33^ CHIS provides a representative sampling of California’s non-institutionalized population through both web and telephone modalities using address-based sampling. Data were collected from March 2021 to November 2022. Depending on respondent preference, the CHIS was offered in English, Chinese (Mandarin or Cantonese), Korean, Vietnamese, or Tagalog. The Vanderbilt University Medical Center institutional review board (IRB) determined that approval was not required because all data were de-identified and publicly available.

### Study Population

This study included 5,744 self-reported Asian respondents aged 18-65 years. We divided the study sample into first and second generation using definitions established in the literature: first generation Asian Americans were defined as respondents who were born outside of the US; and second generation Asian Americans were respondents who were born in the US, but one or both of their parents were born outside the US.^28^ Third and higher generation Asian Americans (i.e., individuals born in the US with both parents born in the US), as well as those who indicated more than one race, were excluded.

### Assessment of ACEs, PCEs, and Psychological Distress

The CHIS assessed 11 conventional^1^ and 4 expanded^2^ ACEs. Conventional ACEs were: 1) household mental illness; 2) household substance misuse; 3) household alcohol dependency; 4) having a family member in prison; 5) parental divorce; 6) domestic abuse; 7) physical abuse; 8) verbal abuse; 9) unwanted sexual touch; 10) coerced to provide sexual touch; and 11) unwanted sexual acts. Expanded ACEs were: 1) parental death; 2) violence in the neighborhood; 3) racism; and 4) living in poverty. We categorized response options as either “yes [including once or more than once]” or “no [including never].” In order to preserve respondent confidentiality, results for three individual sexual abuse ACEs were publicly unavailable. However, these ACEs were included within the composite score of conventional ACEs. ACE scores were recoded as experiencing 0, 1, 2, 3, or ≥4 ACEs, with ≥4 ACEs traditionally used as the cutoff for “many ACEs” due to an elevated risk for adverse health outcomes.^4^ For descriptive purposes, we also included whether respondents were ever formally screened for ACEs by a medical professional outside of CHIS.

The CHIS assessed seven PCEs: 1) able to talk to family about feelings; 2) family stood by respondent during difficult times; 3) feeling safe and protected by an adult in the home; 4) having at least two nonparental adults take interest during childhood; 5) feeling supported by friends; 6) feeling a sense of belonging in high school; 7) enjoying participation in community traditions. Response choices were “always,” “mostly,” “sometimes,” “rarely,” and “never.” Consistent with prior studies,^17^ responses coded as “always” and “mostly” were used to tabulate a composite PCE score, which was then recoded as 0-2, 3-5, and ≥6 PCEs. We set 0-2 PCEs as the cutoff for “lacking PCEs” – which may be associated with worse health outcomes.

Adult mental health was assessed using psychological distress, measured using Kessler’s six-item questionnaire.^34^ The CHIS asked adults the following questions: in the past 30 days, how often did you feel 1) nervous; 2) restless; 3) hopeless; 4) everything was an effort; 5) worthless; and 6) depressed. Response choices for each question were “never,” “rarely,” “sometimes,” “mostly,” and “always,” corresponding to a score of 0 to 4, respectively. Each question was tabulated together to yield an overall psychological distress score with a maximum score of 24. We defined severe psychological distress as a score ≥13, in line with prior studies.^35^

### Covariates

Covariates included in the study were: age in years (18-25, 26-35, 36-45, 46-55, 56-65), sex (male or female), modality of survey (phone or online), household income (<$40,000, $40,000-$80,000, $80,000-$120,000, and >$120,000), health insurance status (yes or no), urbanicity (i.e. residing in an urban environment; yes or no), English proficiency (yes or no), and year of survey (2021 or 2022). The inclusion of income is consistent with similar studies on PCEs.^17,23^ Health insurance status was included because while ACEs do not impact insurance status (nearly all Californians have access to health insurance), insurance status may impact adult mental health. Social capital and networking is associated with improved mental health.^36,37^ In our study, urbanicity acts as a surrogate for Asian American social networks within distinct urban enclaves (such as “Chinatown”). English proficiency likely influences adult mental health; respondents may have worse mental health due to social isolation from lack of proficiency, or better mental health due to residing within a homogenous community that does not necessitate learning English. Finally, the data used in this study were collected between 2021 and 2022. Due to the COVID-19 pandemic, rise in Asian American discrimination, and other major national/world events during this time frame, the inclusion of survey year acknowledges the time-variant effects of these events on adult mental health among survey respondents.

### Statistical Analysis

All statistical analyses were conducted in Stata v18.0 (StataCorp LLC) using survey weights in order to account for nonresponse and noncoverage, ensuring adequate representation. Survey-weighted prevalence estimates for all variables were summarized for first and second generation Asian Americans, and bivariate associations were examined using chi-squared tests. Then, we used generalized linear models with Poisson family regressions and logarithmic linking functions while controlling for covariates to compare the prevalence ratios of ACEs and PCEs between first and second generation Asian Americans. Finally, we ran several generalized linear models to estimate associations between ACEs or PCEs – using composite indices and then by examining each individual ACE or PCE separately – and severe psychological distress in both first and second generation Asian Americans. All regression results are reported as adjusted prevalence ratios (aPR) with 95% confidence intervals (CI).

## RESULTS

Our final study population included 5,744 Asian American respondents aged 18-65 years; 4,307 respondents were identified as first generation and 1,437 as second generation. Among all respondents, 52.0% were female, 42.7% reported an annual income of >$100,000, 93.6% had health insurance, and 97.6% lived in an urban area (Table 1). Second generation respondents were more likely to be younger (75.8% under the age of 35 years, compared to 36.8% of first generation respondents) and be proficient in English (99.6% vs 81.6%). Among all respondents, only 3.3% have ever completed an ACEs assessment with a medical professional; second generation Asian Americans had a slightly greater prevalence (4.6% vs 2.9%).

**Table 1:** Demographic Characteristics^*^ Stratified by Generation Status, CHIS 2021-2022

	Total	First Generation	Second Generation	
	(n=5,744)	(n=4,307)	(n=1,437)	P-value†
Sex				
Male	2,757 (48.0%)	2,104 (49.1%)	653 (45.0%)	0.064
Female	2,984 (52.0%)	2,201 (50.9%)	783 (55.0%)	
Age				
18-25	455 (16.4%)	202 (10.7%)	253 (32.3%)	<0.001
26-35	1,471 (30.6%)	861 (26.1%)	610 (43.5%)	
36-45	1,242 (20.7%)	972 (23.1%)	270 (14.0%)	
46-55	1,403 (18.1%)	1,230 (22.5%)	173 (5.8%)	
56-65	1,173 (14.2%)	1,042 (17.6%)	131 (4.4%)	
Household Income ($)				
<40,000	1,038 (19.2%)	844 (20.1%)	194 (16.5%)	0.030
40,000-80,000	1,137 (21.4%)	861 (22.1%)	276 (19.6%)	
80,000-120,000	1,007 (16.8%)	724 (16.0%)	283 (19.0%)	
>100,000	2,562 (42.7%)	1,878 (41.8%)	684 (44.9%)	
Insured	5,473 (93.6%)	4,078 (92.9%)	1,395 (95.5%)	0.107
Proficient in English	5,013 (86.3%)	3,586 (81.6%)	1,427 (99.6%)	<0.001
Lives in Urban Area	5,531 (97.6%)	4,152 (97.3%)	1,379 (98.3%)	0.035
Ever Completed An ACEs Assessment	174 (3.3%)	111 (2.9%)	63 (4.6%)	0.045

^*^Unweighted raw counts and weighted percent prevalence

^†^P-values estimated using χ2-test

Second generation Asian Americans reported a greater prevalence of ACEs; 65.4% report experiencing ≥1 ACE, compared to 47.5% of first generation Asian Americans (Table 2). Second generation Asian Americans also reported a greater prevalence of ≥4 ACEs (14.0% vs 8.5%), ≤2 PCEs (32.1% vs 22.6%), and severe psychological distress (15.6% vs 5.6%).

**Table 2:** Prevalence^*^ and Adjusted Prevalence Ratios (aPR)^†^ for Reporting ACEs, PCEs and Severe Psychological Distress By Generation, CHIS 2021-2022

	No. (%)*			aPR (95% CI)^†^	
	1st Generation	2nd Generation			
	(*N*=4,307)	(*N*=1,437)	*P*-value^‡^		
≥1 ACEs	2,018 (47.5%)	906 (65.4%)	<0.001	1.20	(1.10, 1.30)
≥4 ACEs	420 (8.5%)	192 (14.0%)	<0.001	1.46	(1.13, 1.88)
≤2 PCEs	1,033 (22.6%)	451 (32.1%)	<0.001	1.51	(1.29, 1.78)
Severe Psychological Distress	231 (5.6%)	168 (15.6%)	<0.001	1.77	(1.32, 2.38)
Conventional ACEs (Included in ACE Score)^§^					
Mental Illness In The Home	397 (8.5%)	276 (21.2%)	<0.001	1.99	(1.57, 2.54)
Experienced Verbal Abuse	1,090 (24.2%)	653 (47.4%)	<0.001	1.57	(1.38, 1.79)
Parents Separated Or Divorced	423 (10.4%)	211 (16.1%)	<0.001	1.24	(0.97, 1.59)
Experienced Physical Abuse	834 (18.7%)	316 (23.6%)	0.003	1.15	(0.95, 1.40)
Alcoholism In The Home	333 (8.4%)	140 (8.8%)	0.725	1.07	(0.79, 1.46)
Witnessed Domestic Abuse	756 (16.3%)	264 (17.7%)	0.364	1.05	(0.85, 1.30)
Drugs In The Home^||^	56 (1.1%)	70 (6.2%)	<0.001	--	
Lived With Anyone Who Served Prison Time^||^	51 (1.1%)	44 (3.3%)	<0.001	--	
Expanded ACEs (Not Included in ACE Score)					
Experienced Racism	990 (21.7%)	665 (42.0%)	<0.001	1.64	(1.43, 1.89)
Witnessed/Experienced Violence In Neighborhood	599 (13.7%)	245 (14.6%)	0.502	1.11	(0.87, 1.42)
Experienced Economic Hardship	887 (22.5%)	282 (20.2%)	0.208	1.08	(0.88, 1.32)
Experienced A Parent Death	891 (20.1%)	189 (10.9%)	<0.001	0.83	(0.64, 1.09)
Lacking PCEs					
Did Not Feel Protected By Adult	849 (20.1%)	347 (25.6%)	0.003	1.31	(1.08, 1.57)
Family Did Not Stand By Respondent	1,327 (31.9%)	603 (43.7%)	<0.001	1.30	(1.15, 1.48)
Unable To Talk To Family About Feelings	2,546 (59.0%)	1,121 (75.9%)	<0.001	1.28	(1.20, 1.37)
Did Not Feel Belonging at School	1,629 (37.6%)	662 (45.5%)	<0.001	1.27	(1.13, 1.43)
Disliked Participating in Community Traditions	2,238 (50.0%)	857 (59.2%)	<0.001	1.18	(1.08, 1.29)
2+ Nonparent Adults Did Not Take Genuine Interest	2,080 (46.7%)	768 (52.4%)	0.009	1.16	(1.06, 1.28)
Did Not Feel Supported By Friends	1,508 (33.0%)	505 (35.3%)	0.240	1.15	(1.00, 1.33)

Abbreviations: *ACE*, adverse childhood experience; *PCE*, positive childhood experience

^*^Unweighted raw counts and weighted percent prevalence

^†^Adjusted prevalence ratios (aPR) were estimated using generalized linear models with Poisson family regressions and logarithmic linking functions while controlling for age category, sex, annual household income, health insurance status, English proficiency, urbanicity, survey year, and survey modality. The results displayed are aPRs for reporting ACEs, PCEs, and psychological distress in second generation Asian Americans, compared to first generation Asian Americans as the reference group

^‡^P-values estimated using χ2-test

^§^Sexual abuse data are not publicly available to protect confidentiality

||Household substance use and family in prison were omitted due to relatively small sample sizes

Among individual ACEs, second generation Asian Americans reported a greater prevalence of experiencing or witnessing household mental illness, household substance misuse, family in prison, parental divorce, physical abuse, verbal abuse, and racism (Table 2). First generation Asian Americans reported a greater prevalence of parental death. Among PCEs, second generation Asian Americans reported a greater prevalence of being unable to talk to family about their feelings, family not standing by the respondent, not feeling protected by an adult at home, nonparental adults not taking interest, not feeling belonging in high school, and disliking participation in community traditions.

When estimating associations between generation status, ACEs, and PCEs, we found that second generation Asian Americans were more likely to report ≥1 ACE (aPR, 1.20; 95% CI, 1.10-1.30), ≥4 ACEs (aPR, 1.46; 95% CI, 1.13 to 1.88) and ≤2 PCEs (aPR, 1.51; 95% CI, 1.29 to 1.78) relative to first generation Asian Americans (Table 2). Among individual ACEs and PCEs, second generation Asians were more likely to report experiencing household mental illness (aPR, 1.99; 95% CI, 1.57 to 2.54), verbal abuse (aPR, 1.57; 95% CI, 1.38 to 1.79), racism (aPR, 1.64; 95% CI, 1.43 to 1.89), a lack of protection from an adult at home (aPR, 1.31; 95% CI, 1.08 to 1.57), family not standing by the respondent (aPR, 1.30; 95% CI, 1.15 to 1.48), an inability to talk to family about feelings (aPR, 1.28; 95% CI, 1.20 to 1.37), a lack of belonging in high school (aPR, 1.27; 95% CI, 1.13 to 1.43), a dislike for participating in community traditions (aPR, 1.18; 95% CI, 1.08 to 1.29), nonparental adults not taking interest (aPR, 1.16; 95% CI, 1.06 to 1.28), and a lack of support from friends (aPR, 1.15; 95% CI, 1.00 to 1.33). Results for household substance use and family in prison were omitted due to a low number of respondents (n=126 and n=95, respectively).

**Figure 1:**
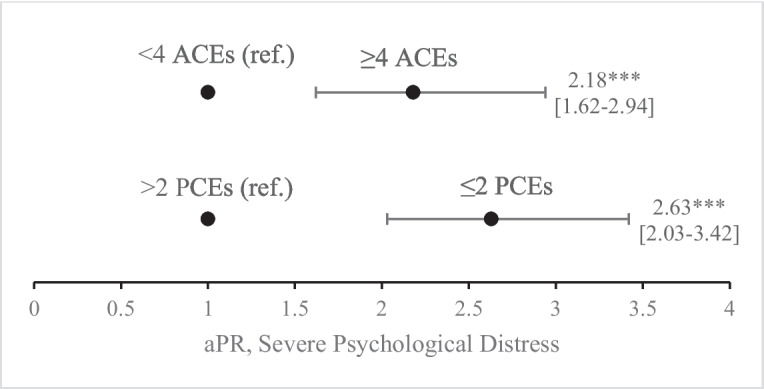
Adjusted Prevalence Ratios (aPR) for reporting severe psychological distress in the presence of ACEs or PCEs†,‡. * *p*<0.05, ** *p*<0.01, *** *p*<0.001. †Data Source: California Health Interview Survey (CHIS) 2021 and 2022. ‡Adjusted prevalence ratios (aPR) were estimated using generalized linear models with Poisson family regressions and logarithmic linking functions while controlling for age category, sex, annual household income, health insurance status, English proficiency, urbanicity, survey year, and survey modality. The results displayed are aPRs for reporting severe psychological distress across all respondents in our study population who experienced ≥4 ACEs or ≤2 PCEs.

**Figure 2:**
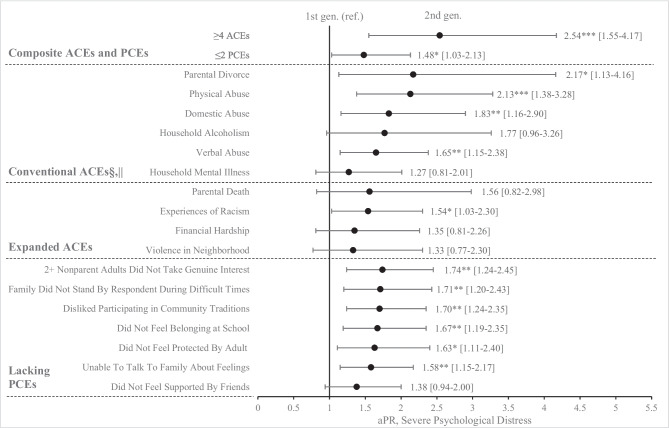
Adjusted Prevalence Ratios (aPR) for severe psychological distress associated with ACEs and PCEs in second generation compared to first generation Asian Americans†,‡. * *p*<0.05, ** *p*<0.01, *** *p*<0.001. †Data Source: California Health Interview Survey (CHIS) 2021 and 2022. ‡Adjusted prevalence ratios (aPR) were estimated using generalized linear models with Poisson family regressions and logarithmic linking functions while controlling for age category, sex, annual household income, health insurance status, English proficiency, urbanicity, survey year, and survey modality. §Sexual abuse data was protected for confidentiality and not publicly available in CHIS. ||Household substance use and family in prison were omitted due to relatively small sample sizes.

When estimating associations between ACEs, PCEs, and severe psychological distress in adulthood, we found that overall, those who experienced ≥4 ACEs or ≤2 PCEs were more likely to experience severe psychological distress (aPR, 2.18; 95% CI, 1.62-2.94 and aPR, 2.63; 95% CI 2.03-3.42, respectively) (Fig. 1). When investigating differences between generations, second generation Asian Americans were more likely to report severe psychological distress compared to first generation Asian Americans (aPR, 1.77; 95% CI, 1.32-2.38) (Table 2). Second generation Asian Americans who experience ≥4 ACEs were more likely to report severe distress (aPR, 2.54; 95% CI, 1.55 to 4.17), compared to first generation Asian Americans who experience ≥4 ACEs (Fig. 2). Second generation Asian Americans who experience ≤2 PCEs were also more likely to report severe distress (aPR, 1.48; 95% CI, 1.03 to 2.13) compared to first generation Asian Americans who experience ≤2 PCEs. When examining childhood experiences individually, second generation Asians were more likely to report severe distress when experiencing parental divorce (aPR, 2.17; 95% CI, 1.13 to 4.16), physical abuse (aPR, 2.13; 95% CI, 1.38 to 3.28), domestic violence (aPR, 1.83; 95% CI, 1.16 to 2.90), verbal abuse (aPR, 1.65; 95% CI, 1.15 to 2.38), racism (aPR, 1.54; 95% CI, 1.03 to 2.30), nonparental adults not taking interest (aPR, 1.74; 95% CI, 1.24 to 2.45), family not standing by the respondent (aPR, 1.71; 95% CI, 1.20 to 2.43), a dislike for participating in community traditions (aPR, 1.70; 95% CI, 1.24 to 2.35), a lack of belonging in high school (aPR, 1.67; 95% CI, 1.19 to 2.35), a lack of protection from an adult at home (aPR, 1.63; 95% CI, 1.11 to 2.40), and an inability to talk to family about feelings (aPR, 1.58; 95% CI, 1.15 to 2.17) compared to first generation Asian Americans experiencing the same events. Results for household substance use and family members in prison were again omitted due to a low number of respondents.

## DISCUSSION

This study explored differences in ACEs, PCEs, and severe psychological distress between first and second generation Asian Americans in California. We found that second generation Asian Americans reported a greater prevalence of negative childhood experiences and a greater prevalence of severe psychological distress. Second generation Asian Americans with many ACEs or few PCEs were more likely to experience severe psychological distress. Second generation Asian Americans were also more likely to experience many of the individual ACEs, and less likely to experience many of the PCEs. Finally, experiencing or lacking many of the ACEs and PCEs, respectively, were individually associated with severe psychological distress in second generation Asian Americans.

Our results demonstrate that second generation Asian Americans reported worse outcomes despite being born, raised, educated, and employed in the US, thus challenging the “model minority” myth that overlooks the unique mental health needs of this population.^38^ The ACEs and PCEs associated with severe psychological distress can be broadly categorized into two themes: second generation children versus first generation parents (e.g. physical and verbal abuse, being unable to talk to family about feelings), and second generation Asians versus non-Asian Americans (e.g. racism, not feeling belonging at school). These differences may reflect the important role that social support structures play in mitigating mental illness,^39^ and how second generation Asian Americans may be uniquely isolated from both their first generation parents, as well as from the general American population.

Language and cultural barriers exist between first and second generation Asian Americans. For example, studies have found that only 40% of second generation Asian Americans are able to speak their parent’s native language; in contrast, this number is closer to 80% for second generation Hispanic Americans.^40^ Furthermore, second generation Asian Americans grow up at the crossroads of family-centered Asian values and individualistic Western values; experiencing these cultural differences has been found to increase the likelihood of intergenerational conflicts during childhood.^41^ Asian American parents are also less likely to worry about mood disorders in their children,^42^ and first generation Asian Americans are less likely to seek help for mental health, possibly due to cultural stigma.^43,44^ Overall, intergenerational differences may lead to conflict that results in verbal and physical abuse, as well as make it difficult for second generation Asian Americans to talk to family about their feelings. Domestic violence and parental divorce may strain an already tenuous support system, further leading to increased psychological distress.

Second generation Asian Americans may also struggle to assimilate with the general American population. Among young Asian American adults, strong peer relationships (i.e. friends at school) have been found to inversely correlate with depression and suicidal ideation.^45^ However, 38% of second generation Asian Americans have felt the need to hide parts of their heritage in order to avoid judgment from peers.^46^ 90% of Asian Americans have experienced discrimination and 78% have been treated as a foreigner, even if born in the US.^47^ While second generation Asian Americans may identify with Western values, these statistics highlight the challenges that Asian Americans face during the process of acculturation.

The immigrant paradox (i.e., first generation immigrants have better health outcomes despite facing great obstacles^48^) is also present in our study, with first generation Asian Americans reporting lower prevalence of negative childhood experiences and adverse mental health outcomes. This paradox may be the result of self-reporting bias. Due to cultural views on mental health,^43^ first generation Asian Americans may be less likely to respond accurately on health surveys. However, it is also possible that first generation Asian Americans had better support in their home country during childhood, as they shared the same culture as their family and peers. Furthermore, first generation respondents may have had better socioeconomic status compared to their peers, giving them the capacity to immigrate in the first place. As a result of possible privileged upbringings, it is possible that first generation Asian Americans truly experience less ACEs and more PCEs. In our study, first generation Asian Americans were well compensated (>40% earned >$100,000 annually), which may support this hypothesis. However, this theory does not take into account refugees of war who experienced trauma in childhood. Thus, more research is needed to examine whether there are associations between country of origin, circumstances of immigration, and mental health.

PCEs have previously been found to attenuate the effects of ACEs as well as independently lead to improved adult health.^17,18,20,22,23^ Our study found that lacking PCEs is associated with severe psychological distress. While this result has not been reported previously, prior studies have shown that a lack of social support is associated with increased morbidity and mortality.^49^ Social support may lead to improved resiliency, which is protective against stressors; in contrast, lacking social support results in a lack of resilience, predisposing individuals to a higher likelihood of psychological distress due to external stressors.^50,51^ Since PCEs are closely related to social support from friends, family, and community, it is plausible that lacking PCEs may lead to distress through similar mechanisms. However, more research must be done at the intersection of ACEs, PCEs, and adult health.

This population-based study had several limitations. We were unable to determine causality between ACEs, PCEs, and adult mental health. This study was limited to California and may not be representative of Asian Americans nationwide. Due to self-reporting bias, the prevalence of ACEs, PCEs, and psychological distress may be underreported. The CHIS measured adult mental health using the Kessler distress scale, and did not ask about specific clinical diagnoses. We were unable to analyze sexual abuse because data were not publicly available. We also did not analyze household substance use and family in prison due to small sample sizes. Third and higher generation Asian Americans were omitted due to small sample sizes. Finally, we were unable to study so-called “1.5 generation” Asian Americans (i.e. individuals born outside the US who immigrated to the US in childhood).

Despite significant intergenerational differences in ACEs, PCEs, and adult mental health, only 3.3% of our study population has ever been screened for ACEs by a clinician. Our study highlights the need for physicians to screen for adverse and positive childhood experiences in order to provide personalized, trauma-informed care in this marginalized population. Screening in childhood can allow providers to intervene by promoting healthy parenting and referring families to social services. In adulthood, screening can identify those at risk for mental illness and identify patients who require access to mental health professionals. These efforts will hopefully lead to improved mental health equity and long-term health outcomes.
